# Mobbing (bullying at work) in Italy: characteristics of successful court cases

**DOI:** 10.5249/jivr.v10i1.945

**Published:** 2018-01

**Authors:** Daniela Acquadro Maran, Silvia Bernardelli, Antonella Varetto

**Affiliations:** ^*a*^Department of Psychology, University di Torino Via Verdi 10 – 10124, Torino, Italy.

**Keywords:** Bullying, Workplace, Compensation, Violent behavior

## Abstract

**Background:**

Mobbing (bullying at work) refers to a form of psychological harassment that occurs in the workplace, in which the victim must be systematically and continuously persecuted for a long period of time. The aim of this work is to analyze the court judgments related to mobbing.

**Methods:**

The data, collected from a website that included judgments from an Italian court, were analyzed according to the literature, identifying the type of victims, consequences, methods of harassment, type of mobbers, and compensation decided by the court.

**Results:**

A total of 35 court sentences were analyzed. The findings showed that the duration of the mobbing campaign was on average 1.5 years and that the frequency of harassment was every day in most cases. In the majority of cases (17, 48.6%) the mobbing occurred in a private company. The gender of the victims who reported the mobbing experience was predominantly female (19, 54.3%), and on average, the victims were 44.54 years of age. The victims were classified as captives (12, 34.3%), scapegoats (8, 22.9%), ambitious (8, 22.9%), passives (5, 14.3%) and hypochondriacs (2, 5.7%). The mobbers were predominantly men (25, 71.4%) and on average 53.20 years of age. They were classified as casual (12, 34.3%), sadists (11, 31.4%), instigators (8, 22.9%) and choleric (4, 11.4%). The witnesses were described in the majority of cases as active, while the asymmetry of power was vertical. On average, the victims suffered 4.9 types of harassment, and the most cited consequences were anxiety disorder and physical symptoms. The motives for beginning the mobbing campaign were principally related to difficulties in relationships. The compensation imposed by the court ranged from less than 20,000 to more than 70,000 euros.

**Conclusions:**

The sentences analyzed showed that for different types of victims, there are behaviors, motives and consequences that are linked to different types of perpetrators.

## Introduction

Mobbing (bullying at work) refers to a form of psychological harassment that occurs in the workplace, in which the victim must be systematically and continuously persecuted for a long period of time. ^1^ Mobbing is a phenomenon that affects a large number of workers: Samnani and Singh ^2^ reported a prevalence rate of nearly 50% in the U.S., 4% in Italy and 5% to 10% in Europe. 

Ege^3^ described a method for identifying a mobbing situation. This method consists of seven parameters, which are objective and scientifically verifiable, that need to be taken into account for a conflict to be considered mobbing. First of all, the conflict has to occur only in the workplace and has to occur every day, a few times every week or a few times every month for at least 6 months. The mobbing campaign is characterized by action against free speech and/or systematic isolation, a change of tasks, attacks on reputation and violence or threats thereof. Moreover, the victim cannot properly defend him/herself because there is an asymmetry of power. This asymmetry can be horizontal (the victim and the mobber are in the same organizational position/role, but the victim perceives him/herself to be weaker than the mobber, or vice versa), vertical (the mobber or the victim has a higher position/role), or strategic (the manager enacts the mobbing campaign to exhaust the victim to oust him/her from the workplace).^3^

Three actors are involved in a mobbing situation: the victim, the mobber, and the witness. 

The definition of a victim can be explained based on the following five categories: captive (the victim is able to recognize the phenomenon but does not know effective strategies to prevent it from happening); passive (the victim is affable, servile, and incapable of saying “no”); ambitious (the victim works to keep high levels of effectiveness and efficiency and elicits envy from colleagues); hypochondriac (the victim tells anyone about his/her uneasiness, tends to feel depressed, and is always dissatisfied); and scapegoat (the victim is weak, and his/her colleagues vent their anger towards him/her). As consequences of mobbing campaign the victim can suffer psychosomatic (e.g., sleeping difficulties), emotive (e.g., frustration) and/or physical problems (e.g., cardiovascular diseases).^4,5^ The victim can develop psychological problems such as mood disorders, anxiety disorders, or burnout. The most frequent diagnosis is adjustment disorder.^6^

 The mobber is "the executioner", the individual who starts and applies the mobbing behaviors. 

Safina and Podgornaya^7^ conducted research and showed that there are different types of mobber goals ^8^ in the work harassment campaign: 

- to diminish the victim's self-esteem and feeling of power by, for example, spreading rumors and malice to attack the victim; 

- to create emotional distress and feeling such as anger towards another person;

- to gain power, authority, higher status, respect (the mobber has a distorted view of him/herself, as he/she considers him/herself to be superior and thinks that he/she is allowed to become angry; this motive is associated with violence against other colleagues who are perceived as being inferior); 

- to test a fresher destruction strategy (he/she hurts another person for the sake of doing it and is not inclined to let the victim escape);

- to drive out a worker (the victim) (he/she is dissatisfied with his/her life and with other colleagues and creates an unsatisfactory and relatively tense climate); and

- to progress up the career ladder (he/she tries to make his/her way in the organization using all possible means). 

According to Ege,^3^ these mobbers can be referred to as an instigator, choleric, a megalomaniac, a sadist, frustrated, and a careerist, respectively. Furthermore, Leymann^9^ identified motives for mobbing: the lack of rule observance, punishment, the removal of a worker, difficulty in relationships. ^10^ The harassment of and discrimination against a worker due to political or religious beliefs are described as mobbing. ^11^ In addition, the rejection of a sexual advance can be the beginning of a mobbing campaign.^12^

Witnesses can be classified as active or passive. Active witnesses take part in the phenomenon directly, thereby sustaining it. Passive witnesses are not directly involved in the persecution, but even if they know about it, they do not help the victim.^3,11^

**Current study**

The aim of this work is to analyze the court judgments related to mobbing in a descriptive way. Knowing how the phenomenon is punished permits victims and organizations to pay attention to behaviors that represent workplace harassment: for victims, a cause of mobbing represents a cost; for an organization, mobbing can damage its image. Moreover, for victims and organizations, mobbing has economic consequences, e.g., the medical exam for victims and early retirement payments for organization.^13^

## Methods 

The data were collected from a website that included judgments from an Italian court. The sentences were published in accordance with the Italian law on privacy. Therefore, the court might have omitted some information about the actors who were involved in the case (e.g., name and surname). This website was well known in Italian public administration and was devoted to training and updating lawyers in the private and public sectors. The inclusion criterion of the judgments in this research was as follows:

i. the judgment recognized a victim of mobbing and the physical and emotive consequences of the victimization; and

ii. the court handed down a sentence for an offence related to mobbing.

Cases in which the judgment was in favor of the victim but the references were principally in favor of other phenomena were excluded. Since, for Italian law, mobbing is not a crime, the accuser’s lawyer could refer to mobbing and behaviors associated with the phenomenon that are punishable: for example, having caused health problems, defamation, and sexual harassment. At the same time, the defense attorney could refer the behavior to a relational incompatibility. The court could ask the opinion of experts in the field and consequently issue a ruling for mobbing or other harassment behaviors, such as straining or occupational stalking. These cases were excluded. 

A database was built according to the boundaries and characteristics of the phenomenon described in the literature: 

- the duration of the mobbing campaign (in years) (one item), the frequency of the harassment (possible responses: every day, a few times every week or a few times every month) (one item) and the workplace (one item); 

- the type of mobber: instigator, choleric, megalomaniac, sadist, frustrated, or careerist (yes/no responses) (6 items);

- the type of victim: captive, passive, ambitious, scapegoat, or hypochondriac (yes/no responses) (5 items);

- witnesses: active viewers (yes/no) (one item) and passive viewers (yes/no) (one item);

- asymmetry of power: horizontal, vertical, or strategic (yes/no response) (3 items);

- behaviors: actions against free speech, systematic isolation, a change of tasks, attacks on reputation, threats, or physical assault (yes/no response) (six items);

- motives: failure to comply with the rules, punishment, expulsion, discrimination, requests for sexual relationships, or difficult relationships (yes/no response) (six items); 

- consequences: mood disorders, anxiety disorders, adjustment disorder, post-traumatic stress disorder, emotive problems, or physical problems (yes/no response) (six items); and

- compensation imposed by the court: less than 20,000 euros, 21,000-50,000 euros, 51,000-70,000 euros, or more than 71,000 euros.

**Procedure**

The website was visited in June 2016. Of the 47 sentences, 35 were included in the present study. Of the 12 sentences that were excluded, two were not in favor of the victim, and 10 were cases that referred to the sentence with the first degree of judgment and for which there were available sentences that referred to the second degrees of judgment (included in the 35 sentences analyzed in this study). The sentences that were included were read by two students who were trained by the authors and who received credits for this activity. Their purpose was to enter all the information into a database. In the next phase, all the information that was entered was checked by the researcher, and adjustments were made. The methodology of content analysis was used to process the categorization of victims and perpetrators. As suggested by Annese and Mininni^14^, the results were examined by each author independently; the authors then jointly discussed the meaning attributed to the data until an agreement on the results was reached. For example, in one case, the typology of the victim was categorized in different ways, and the discussion led to a decision. Consistency was guaranteed by reproducibility (or intercoder reliability). Descriptive statistics were calculated using IBM SPSS Statistics, version 22. Descriptive measures (mean ± SD) were calculated for all variables. Correlations were calculated to examine the relationship between the number of harassment behaviors and consequences and between the number of consequences and compensation.

## Results

The analysis of the sentences showed that the duration of the mobbing campaign varied from 1 to 4 years, with an average of 1.5 years (SD = 1). The frequency of harassment was every day in 34 of the cases. In one case, the frequency was a few times every week. In the majority of cases, the workplace was a private company (17, 48.6%). In five cases (14.3%), the mobbing campaign occurred in an educational institution (Table 1). In five cases (14.3%), it occurred in a health institution. In three cases (8.6%), the mobbing campaign occurred in a police or military force. In two cases (5.7%), it occurred in public administration. In another two cases (5.7%), the mobbing campaign occurred in show business. In one case (2.9%), it occurred in a sports club. 

Overall, 16 (45.7%) victims were men, whereas 19 (54.3%) were women. The age of the victim was indicated in 11 cases. On average, the victim’s age was 44.54 years (range: 20-67; SD = 10.15). Most of them were classified as captives (12, 34.3%). However, eight victims (22.9%) were classified as scapegoats, eight (22.9%) as ambitious, five as passives (14.3%) and two (5.7%) as hypochondriacs. Regarding the mobbers, 10 (28.6%) were women, whereas 25 (71.4%) were men. In six cases, the mobber’s age was indicated. 

On average, the mobber’s age was 53.20 years (range: 35-72; SD = 11.10). Most of them were classified as casual mobbers (12, 34.3%), although 11 mobbers (31.4%) were classified as sadists, eight (22.9%) as instigators and four (11.4%) as choleric. The witnesses were described in 15 cases (42.9%) as active, as they actively participated in the mobbing campaign. In nine cases (25.7%), they were described as passive. In 11 cases, there was no description of the witnesses. The asymmetry of power was classified in most of the cases as vertical (29, 82.9%). In four cases (11.4%), the mobbing campaign occurred between peers. In three cases (8.6%), it was a strategy that was adopted by the organization to oust the worker. 

The mobbing campaigns were characterized by several behaviors. On average, the victims suffered 4.9 different types of harassment (range: 3-6). Actions against free speech were cited in 34 (97.1%) of the cases. In 30 cases (85.7%), a change of tasks was cited. In 26 cases (74.3%), an attack on reputation occurred. In 24 cases (68.6%), systematic isolation was reported. In 24 cases (68.6%), an assault occurred. In 20 cases (57.1%), a threat was cited. The motives that were ascribed to the mobbing campaign included relationship difficulties (11, 31.4%), punishment (8, 22.9%), expulsion (8, 22.9%), failure to comply with the rules (6, 17.1%), a request for a sexual relationship (4, 10.1%) and discrimination (2, 5.7%). In four cases, the sentences described two motivations. In two cases, they were punishment and expulsion. In one case, they were relationship difficulties and expulsion. In another case, they were punishment and expulsion. The mobbing campaigns undermined the victims’ well-being. On average, there were 3.71 different types of consequences described by the victims. Most of the victims reported an anxiety disorder (25, 71.4%) and physical problems (25, 71.4%). Mood disorders and emotive problems were each reported by 15 victims (42.9%) and adjustment disorders by 11 (31.4%), whereas post-traumatic stress disorder was not cited. As a consequence of the mobbing campaigns, the court awarded compensation to the victims. In most cases (21, 60%), the compensation was 51,000-70,000 euros. In seven cases (20%), it was more than 71,000 euros. In six cases (17.1%), it was 21,000-50,000 euros, whereas it was less than 20,000 euros in one case. 

**Comparison of the different types of victims:** The analysis of the data from the sentences showed that there were differences among the victims, their mobbers, the type of mobbing, behaviors, motives, consequences and compensation (see Table 1). Concerning gender, captive victims were more often female, while passive victims were generally male. Captive victims were victims of all types of mobbers, while other types of victims were not. Regarding perpetrators, only the instigator was indicated among all types of victims, particularly with victims categorized as ambitious. The casual mobber was indicated more often in the sentences in which the victim was a scapegoat while the sadist in those in which the victims was passive. For the majority of victims, the sentences indicated actions against free speech as the behavior adopted by the mobber. Other common behaviors were isolation and a change of tasks, while attacks on reputation and threats affected captive victims more. Concerning motives, expulsion and relationship difficulties were indicated in the sentences for all types of victims, with different percentages. For one-third of the captive victims, the motive of the mobbing campaign was the denial of a request for a sexual relationship. Anxiety disorders and physical problems were the consequences indicated most often by the majority of victim types, while most passive victims indicated the emotive problems. In most sentences, compensation was indicated as being in the range of less than 20,000 euros and 50,000 euros. In eight sentences, the compensation was less than 70,000 euros, mostly for captive victims. Only two victims (one captive and one ambitious) received more than 70,000 euros.

**Table 1:** Nature and characteristics of mobbing on the basis of type of victims.

	Types of Victims				
	Captive n = 12 n(%)	Scapegoat n = 8 n(%)	Ambitious n = 8 n(%)	Passive n = 5 n(%)	Hypochondriac n = 2 n(%)
**Gender:**					
- Male	4(33.3)	3(37.5)	4(50)	4(80)	1(50)
- Female	8(66.7)	5(62.5)	4(50)	1(20)	1(50)
**Workplace:**					
- Private Company	7(58.3)	2(25)	4(50)	3(60)	1(50)
- Educational Institution	0	3(37.5)	1(12.5)	0	1(50)
- Health Institution	2(16.7)	2(25)	1(12.5)	0	0
- Police/Military Force	1(8.3)	0	1(12.5)	1(20)	0
- Public Administration	1(8.3)	1(12.5)	0	0	0
- Show Business	1(8.3)	0	0	1(20)	0
- Sport Club	0	0	1(12.5)	0	0
**Mobber:**					
- Casual	5(41.7)	4(50)	2(25)	1(20)	0
- Sadist	3(25)	3(37.5)	2(25)	3(60)	0
- Instigator	1(8.3)	1(12.5)	4(50)	1(20)	1(20)
- Choleric	3(25)	0	0	0	1(20)
**Presence of witness:**					
- Active	4(33.3)	4(50)	4(50)	2(40)	1(20)
- Passive	4(33.3)	2(25)	2(25)	1(20)	0
**Asymmetry:**					
- Horizontal	2(16.7)	0	0	2(40)	0
- Vertical	9(75)	8(100)	7(87.5)	3(60)	2(100)
- Strategical	1(8.3)	1(12.5)	2(25)	0	0
**Behaviors:**					
- Actions against free speech	12(100)	8(100)	8(100)	5(100)	1(50)
- Isolation	6(50)	7(87.5)	6(75)	3(60)	2(100)
- Change of tasks	10(83.3)	6(75)	8(100)	4(80)	2(100)
- Attack on reputation	10(83.3)	6(75)	6(75)	3(60)	1(50)
- Threat	8(66.7)	5(62.5)	5(62.5)	1(20)	1(50)
- Physical assault	9(75)	3(37.5)	6(75)	4(80)	2(100)
**Motives:**					
- Failure to comply with rules	1(8.3)	0	4(50)	1(20)	0
- Punishment	1(8.3)	4(50)	1(12.5)	2(40)	0
- Expulsion	3(25)	1(12.5)	1(12.5)	2(40)	1(50)
- Discrimination	0	0	1(12.5)	1(20)	0
- Request for sexual relationship	4(33.3)	0	0	0	0
- Relationship difficulties	2(16.7)	4(50)	2(25)	1(20)	2(100)
**Consequences:**					
- Mood disorders	6(50)	3(37.5)	4(50)	2(40)	0
- Anxiety disorders	10(83.3)	8(100)	4(50)	1(20)	2(100)
- Adjustment disorder	1(8.3)	6(75)	1(12.5)	1(20)	2(100)
- Emotive problems	4(33.3)	3(37.5)	4(50)	4(80)	0
- Physical problems	10(83.3)	6(75)	4(50)	3(60)	2(100)
**Compensation:**					
- less than 20,000 euros	3(25)	2(25)	0	2(40)	0
- 21,000-50,000 euros	2(16.7)	6(75)	5(62.5)	2(40)	1(50)
- 51,000-70,000 euros	6(50)	0	2(25)	1(20)	1(50)
- more than 71,000 euros	1(8.3)	0	1(12.5)	0	0

## Discussion

Overall, the findings showed that in the sentences, the mobbing parameters as described in the literature^1,2,3^ were respected: there were described cases in which different types of harassment (on average, less than five) were repeated for a long period of time (on average, more than one year), with a frequency of every day for most of the cases in the presence of witnesses (generally active witnesses). In every sentence, the workplace was indicated, with a prevalence of private companies. The prevalence of female victims confirms that women – as in other types of violence – are more prone to report the experience of harassment. All types of victims and perpetrators indicated by the literature^3,7^ were utilized to define the actors as described in the sentences, with a slightly larger presence of captive victims than others. Among the perpetrators, there was a prevalence of casual mobbers and sadists. The asymmetry of power was described in the majority of cases as vertical, confirming the findings by Marinoni and colleagues:^16^ thus, the asymmetry of power was due not only to a discrepancy of power perceived by the victims and the perpetrator but also to their different hierarchical roles. The behaviors that characterized the mobbing campaign were various; the most common were related to psychological harassment (e.g., actions against free speech), confirming the findings from the investigations by Leymann^9^ and Elliot and Davenport.^10^ Physical assault affected all types of victims, once again showing how mobbing behavior includes violent behavior.^17^ Concerning the motives for beginning the mobbing campaign, most of the sentences indicated relationship difficulties, as described in the literature.^9,10^ Other motives, such as discrimination for religious or political reasons, were indicated in only two sentences, while sexual harassment was indicated in four and referred only to captive victims. The consequences of the mobbing campaign in our sample principally involved anxiety disorder, partially disconfirming the results from the investigation by Plopa, Plopa and Skuzinska,^6^ who suggested that the principal consequence was adjustment disorder. On the other hand, physical problems were indicated in most of the sentences, as shown in the investigations by da Silva Joao and Portelada^4^ and Courcy, Morin and Madore.^5^ The compensation decided by the court varied from less than 20,000 to more than 70,000 euros; the higher levels of compensation were attributed to captive victims. Regarding the different types of victims, the findings showed that the method of harassment can differ and that the mobber can have different characteristics. That is, there are not only one type of mobber and one type of victim. For example, captive victims had the same probability of confronting all types of mobbers who harassed through more various behaviors, including sexual harassment, as other types of victims. 

Overall, filing a lawsuit implies a remarkable effort, adding distress to the victims. The victims must spend time searching for communications (e.g., e-mails) and witnesses (e.g., colleagues or ex-colleagues) to produce evidence of their state of psychological and physical illness, locate a counseling center and/or a therapist to certify that the distress is a consequence of mobbing, and find a lawyer. For a victim, it can be difficult to find the strength to fight the mobber and/or the organization, which considers him/herself or itself to be stronger. 

This is a crucial point in intervention for individuals. Victims need to recognize their perception of vulnerability and enhance their ability to defend themselves. A psychoeducational approach can be useful in reducing the guilt – experienced by every type of victim of violence^15^ –associated with a mobbing campaign. The support of a counselor and/or a therapist is important for victims to recognize that the problem is in the workplace and that the victim, the mobber, and the witnesses are actors in an organization that allows the phenomenon. According to Giorgi and colleagues,^18,19^ some organizational factors (e.g., a lack of leadership, climate factors and the interaction between leadership and dynamism) can be antecedents that affect mobbing in the workplace. Consequently, the workplace is the setting in which the phenomenon has to be prevented. In the literature, there are several examples of good preventive practices. Taino, Battaglia, and Imbriani,^20^ for example, suggested the use of assessments for work-related stress as a framework for addressing all organizational risk factors related to relationships and conflicts in the workplace. Dillon^21^ suggested creating and sustaining a positive work culture in which workers are respected, good work is recognized and conflict is dealt with as soon as it arises. 

These interventions must be tolerated on the basis of the type of organizational context in which the mobbing occurred. It is necessary to distinguish between the organization (private companies) and the institution (public administration). In a company that produces goods or services, mobbing involves the victim and his/her family, the mobber, the witnesses, and the organization as actors. In health and educational institutions, the secondary victims of the mobbing could be users (patients in the case of hospitals and students in the case of schools). Patients and students are at a sensitive stage in their lives. In the first case, it is a matter of disease. In the second case, it is a matter of being in training that is not only cultural, as teachers are also mentors.^22^ Additionally, teachers contribute to personal growth and the sphere of values. If health care professionals and teachers are mobbing victims, then the consequences also spill over into the quality of their work, creating a difficult or tense climate. There is the risk of perpetrating the perception of victimization and creating an environment that is affected by violence. Therefore, in the case of hospitals and educational institutions, it is necessary to plan a more widespread intervention that involves the users, as they might suffer from the mobbing campaign indirectly.

Some limitations of this research should be mentioned. The first is the small number of cases that were analyzed. The sample might not have been representative of the population of mobbing victims. Moreover, the sample was decomposed into smaller groups; thus, it was not possible to use more sophisticated data. Future researchers need to expand the sample, utilizing more sites that are dedicated to this issue or accessing court archives. Second, the details of the mobbing campaign were described in some cases but not in others. The different levels of description did not permit in-depth analysis, for example, of the correlation between the behaviors (type, frequency) and the compensation imposed by the court. More information might be useful to characterize the cases more accurately to give a deeper explanation of the phenomenon. 

## Conclusion

The analysis of court sentences related to mobbing showed that it is important to know about the phenomenon and the characteristics of cases (the victim and perpetrator typologies, behaviors, motives for beginning the mobbing campaign, etc.) that are not resolved within organizations with the intervention of a counselor, for example. These cases showed that for different types of victims, there are behaviors, motives and consequences that are linked to different types of perpetrators. The phenomenon is within the organization; thus, prevention in organizations is important to ensure the well-being of workers, to perform conflict management, and to preserve the organization’s reputation and public image and, in the case of health or educational institutions, the users’ well-being. We considered this work to be a first study to know more about the phenomenon. It is a first step in the development of a set of information that individuals and organizations can use to improve their ability to prevent and intervene in mobbing cases. 
